# How tomato leaves stay green: the role of SlGRAS17 and its partners

**DOI:** 10.1093/plphys/kiag155

**Published:** 2026-03-19

**Authors:** Deeksha Singh

**Affiliations:** University of California Davis, Davis, United States

In plants and other photosynthetic organisms, chlorophylls are the primary photosynthetic pigments responsible for light absorption during photosynthesis and impart the green appearance of plants. Plants possess different types of chlorophyll pigments that perform distinct functions. For example, chlorophyll *a* is a core component of the reaction centers of both PSI and PSII, where it absorbs light energy, becomes excited, and transfers an electron to a primary electron acceptor, thereby initiating the photosynthetic electron transport chain. In contrast, chlorophyll *b* is found in the light-harvesting (antenna) complexes, where it captures light energy and transfers it to chlorophyll *a* in the reaction center.

The chlorophyll biosynthetic pathway comprises a series of enzymatic reactions that convert glutamate into 5-aminolevulinic acid (ALA). ALA subsequently undergoes several modification steps, ultimately leading to the formation of protoporphyrin IX (Proto IX). Magnesium chelatase then catalyzes the insertion of Mg^2+^ into Proto IX, generating Mg-protoporphyrin IX, a key intermediate in chlorophyll synthesis (Adhikari et al. 2011). This intermediate is further processed to form chlorophyll *a*, which can subsequently be converted into chlorophyll *b* through the action of chlorophyllide *a* oxygenase (CAO) (Brzezowski et al. 2015).

Chlorophyll biosynthesis is controlled by nuclear-encoded genes, most of which encode enzymes and regulatory proteins. Among these*, GOLDEN2-LIKE* (*GLK*) transcription factors and members of the GATA family play crucial roles in hormone- and light-mediated regulation of chlorophyll accumulation (Zubo et al. 2018; Kim et al. 2023). Several hormone-responsive transcription factors, HY5 (ELONGATED HYPOCOTYL5), and MYBs are known to influence chlorophyll biosynthesis (Zhang et al. 2024). Chromatin immunoprecipitation studies indicate that GLK directly binds to the promoters of chlorophyll biosynthesis genes (Tu et al 2022). However, analyses of mutants lacking these transcription factors reveal the accumulation of residual chlorophyll, suggesting that additional regulatory factors are involved in controlling chlorophyll biosynthesis. To identify genes regulating chlorophyll biosynthesis in tomato, the authors conducted an EMS mutagenesis screen and isolated an *hcm1* loss-of-function mutant. Chlorophyll measurements in *hcm1* leaves revealed increased chlorophyll accumulation compared to wild type (WT). To pinpoint the mutation responsible for altered greening, a mapping-by-sequencing approach (Garcia et al. 2016) was employed. This analysis identified a single nucleotide polymorphism (SNP), a G-to-A substitution, in the GRAS family gene *SlGRAS17* (Solyc02g085340), leading to premature termination of the SlGRAS17 protein. To further validate the role of SlGRAS17 in chlorophyll biosynthesis, CRISPR-mediated knockout mutants were generated. The 2 homozygous mutants, *gras17-cr8* and *gras17-cr14*, exhibited elevated levels of chlorophyll *a* and *b* compared with WT, whereas overexpression of *SlGRAS17* produced a pale-green phenotype with a significant reduction in chlorophyll accumulation.

To elucidate the mechanism by which SlGRAS17 regulates greening, the authors analyzed its subcellular localization and observed that SlGRAS17 is localized in the nucleus. The authors then performed qPCR to examine the expression of 12 photosynthesis-associated genes in WT plants, *Slgras17* knockout mutants *gras17-cr8*, and *SlGRAS17* overexpressing lines (*SlGRAS17-OE2*). The analysis revealed that the expression of 8 chlorophyll biosynthesis target genes was significantly upregulated in *gras17-cr8* but conversely downregulated in *SlGRAS17-OE2*. Additionally, *GLK1* expression was also increased in *gras17-cr8* and reduced in *SlGRAS17-OE2*. Since *GLK1* may be a target of SlGRAS17, the authors showed that SlGRAS17 directly binds to the *GLK1* promoter using yeast 1-hybrid (Y1H) assays. To further explore their genetic interaction, virus-induced gene silencing (VIGS) of *GLK1* was performed in WT and *gras17-cr8* backgrounds. In contrast to the enhanced chlorophyll levels observed in *gras17-cr8*, silencing *GLK1* in the *gras17-cr8* mutant suppressed chlorophyll accumulation to levels comparable to silenced WT plants, suggesting that SlGRAS17 regulates greening by repressing *GLK1* expression.

Transcriptional regulation in plants is highly complex, often involving interactions between transcription factors and various cofactors. GRAS proteins function by forming homodimers or heterodimers. To identify potential interacting partners of SlGRAS17, a yeast 2-hybrid (Y2H) assay was performed, revealing that SlSEUS3 (Solyc06g059750, *SlSEU3*), a member of the LIM domain-binding family, interacts with SlGRAS17. Previous studies have shown that SEU proteins associate with LEUNIG (LUG) to function as transcriptional co-repressors (Sridhar et al. 2004; Grigorova et al. 2011). Although no direct interaction was detected between LUG and SlGRAS17, SlSEU3 was found to interact with both SlGRAS17 and LUG, suggesting that SlSEU3 serves to bridge the formation of a ternary complex.

To investigate the genetic network underlying SIGRAS17 function, *lug* knockout lines were generated and found to exhibit enhanced greening, whereas *SILUG* overexpression lines displayed pale-green leaves. These phenotypes closely resemble those observed in *Slgras17* mutants and *SlGRAS17* overexpression lines, respectively. Consistent with these observations, *GLK1* transcript levels were elevated in *lug* mutants and reduced in *SlLUG* overexpression lines, supporting a role for SlLUG in repressing *GLK1* expression. Importantly, genetic interaction analysis revealed that the *lug/SlGRAS17-OE2* line exhibited a dark-green phenotype similar to the *lug* mutant and stronger than *SlGRAS17-OE2* alone, indicating that the *SlGRAS17* overexpression phenotype is dependent on SlLUG function. Collectively, Wang et al. 2026 showed that SlLUG is essential for SlGRAS17-mediated repression of chlorophyll accumulation, with SlGRAS17 relying on the SlSEU3–SlLUG complex to suppress *GLK1* expression and finely regulate chlorophyll levels in tomato (Fig. 1). This work enhances our understanding of the molecular components underlying chlorophyll biosynthesis in tomato and provides a framework for developing key agronomic traits

**Figure 1:**
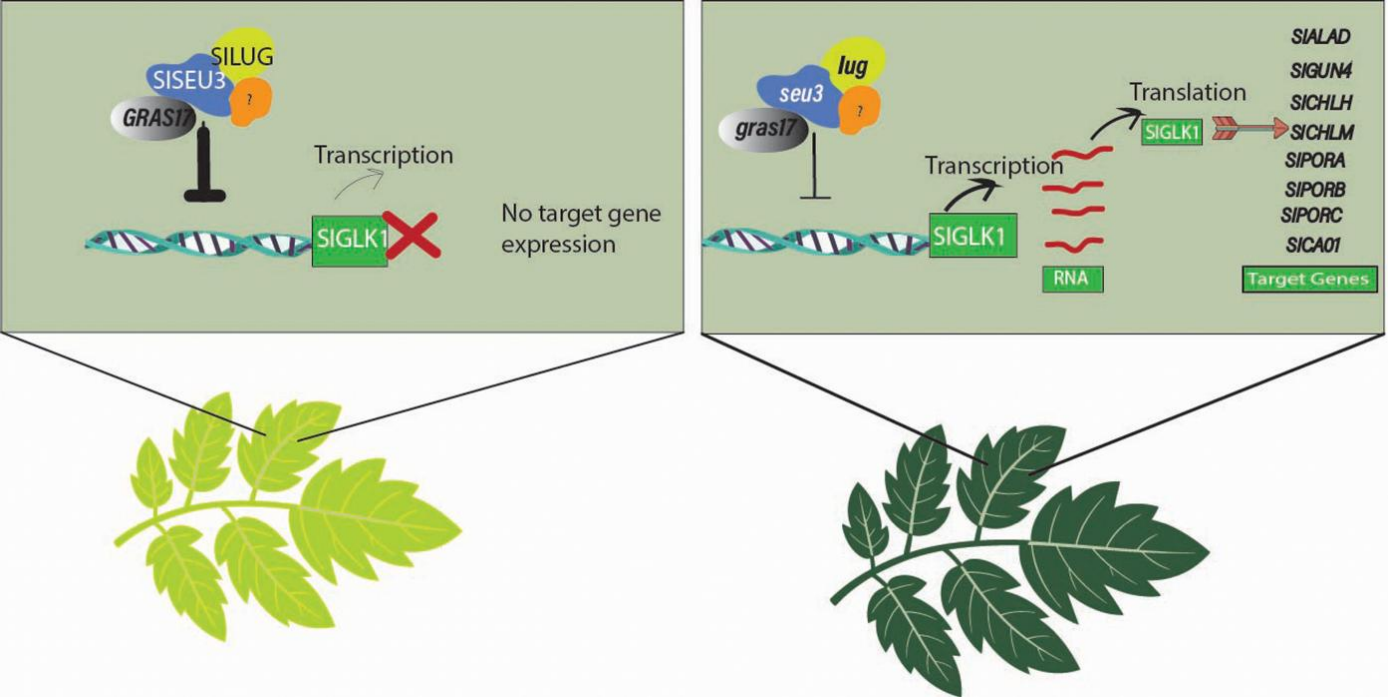
A proposed working model SlGRAS17 interacts with SlSEU3 and SlLUG to form a repressor complex that inhibits *SlGLK1* expression and its downstream targets, leading to reduced chlorophyll biosynthesis and a pale-leaf phenotype. In contrast, *gras17* and *lug* mutations relieve this repression, resulting in enhanced *GLK1* expression and activation of multiple *GLK1* target genes, thereby promoting chlorophyll accumulation and leaf greening.

## Data Availability

The data that support the findings of this study are available from the author upon reasonable request.
